# Pericardial Cyst, A Rare Incidental Finding in an Uncommon Location: A Case Report

**DOI:** 10.7759/cureus.26037

**Published:** 2022-06-17

**Authors:** Muhammad Atif Masood Noori, Mahsa Mohammadian, Hasham Saeed, Rachel Abboud, Andrew Polyak, Qirat Jawed, Dhaval Shah, Prabhjot Singh, Marina Ibrahim, Meherwan Joshi

**Affiliations:** 1 Internal Medicine, RWJBarnabas Health/Trinitas Regional Medical Center, Elizabeth, USA; 2 Cardiology, St. Joseph's University Hospital, Paterson, USA; 3 Cardiology, St. Joseph’s University Hospital, Paterson, USA; 4 Internal Medicine, St. George’s University, Elizabeth, USA; 5 Cardiology, RWJBarnabas Health/Trinitas Regional Medical Center, Elizabeth, USA

**Keywords:** pericardial cyst management, symptomatic pericardial cyst, incidental pericardial lesion, pericardial lesion, pericardial cyst

## Abstract

A pericardial cyst is one of the rare causes of mediastinal masses. Most of the cases are secondary to congenital incomplete fusion of the pericardial sac. More than two-thirds of the cases are present in the right cardiophrenic angle, and the left cardiophrenic angle is the second most common location. In our study, we illustrated an incidental finding of the pericardial cyst in a patient who presented with nonspecific symptoms and was found to have a left-sided cardiophrenic pericardial cyst, which is only found in about 20% of the cases. A CT scan and echocardiogram confirmed the diagnosis of a 4.39-centimeter cyst with no signs of complications like tamponade or pericarditis. As the patient's symptoms resolved, outpatient follow-up with serial echocardiogram was advised. Through this report, we aim to raise awareness of the importance of further investigation for nonspecific symptoms like atypical chest tightness and differentiating simple pericardial cysts from other pericardial lesions. Based on the symptoms, size, and compression effect of the cyst, management may vary from serial echocardiogram to aspiration or surgical resection.

## Introduction

A pericardial cyst (PC) is a rare incidental finding with an incidence of about 1 in 100,000 patients and makes up about 7% of all mediastinal masses [1]. First discovered as an incidental finding in an autopsy [2], PCs are rare intrathoracic structures that occur most commonly due to incomplete fusion in embryogenesis, leading to herniation or weakness in the pericardial sac, forming a diverticulum. This outpouching can either persist as a pericardial diverticulum or form a PC when the communication to the pericardial sac becomes obliterated. The interrelationship between the pericardial diverticulum and cyst sharing a common embryonic origin was first recognized by Rohn A in 1903 [3]. The pericardial cyst can also be acquired as a complication of pericarditis or inflammatory conditions and surgery. Histologically, they are fluid-filled cavities that are lined with mesothelial cells and attach to the parietal pericardium but do not communicate with the pericardial space. Typically found in the third or fourth decade of life, PCs have no gender predilection in their prevalence [4]. These are commonly located in the right cardiophrenic triangle (70%) followed by the left cardiophrenic triangle (22%) and, finally, other parts of the mediastinum (8%) [5]. We present a middle-aged man who presented with stroke-like symptoms and atypical chest pain and was found to have a pericardial cyst.

## Case presentation

**Figure 1:**
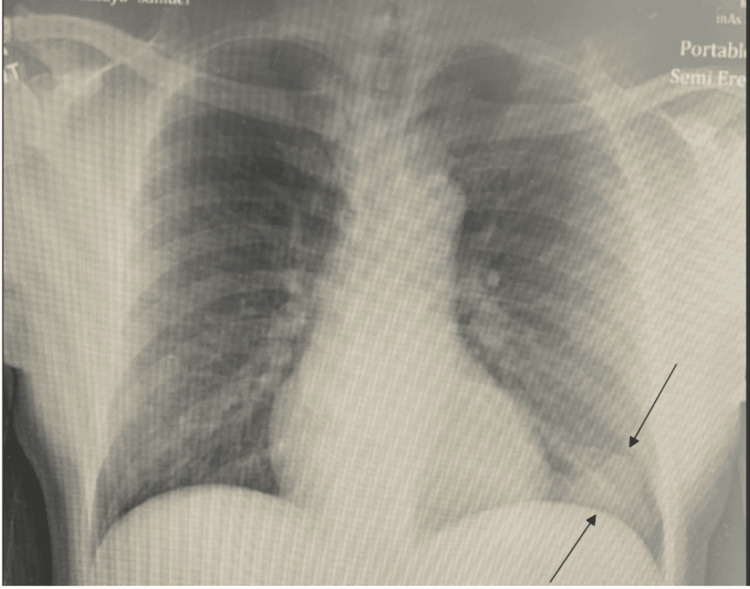
CXR showing pleural-based density measuring 4.1 cm (black arrows) CXR: chest X-ray

A 47-year-old male with a past medical history of hypertension presented to the emergency department with complaints of numbness and tingling in his left big toe that started suddenly while he was sitting at home. He also reported one episode of non-exertional chest tightness that lasted for one minute and resolved spontaneously. A review of systems was otherwise unremarkable. On arrival, the patient’s temperature was 98.1°F, pulse was 82/min, respiratory rate was 18/min, and blood pressure was 186/113 mmHg. Electrocardiogram demonstrated normal sinus rhythm. A computed tomography (CT) scan of the head was unremarkable. CT angiography of the head and neck was also unremarkable for any significant vascular occlusion. However, a chest X-ray (CXR) showed a pleural-based density measuring 4.1 cm (Figure 1).

**Figure 2:**
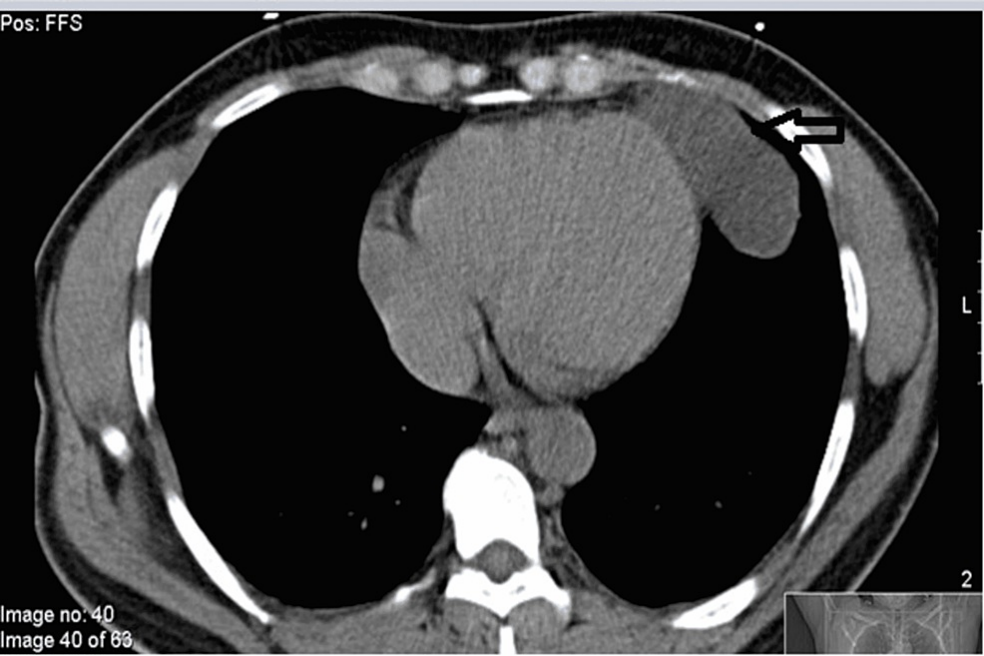
CT chest showing pericardial cyst measuring 8.1 x 4.2 cm (black arrow)

To further evaluate the pleural-based density, a CT scan of the chest was performed, which showed an 8.1 x 4.2 cm homogenous density in the left anterior cardiophrenic angle suggestive of a pericardial cyst (Figure 2). A transthoracic echocardiogram was performed, which showed an ejection fraction of 55-50% with no evidence of thrombus. It further confirmed the presence of a 4.39 cm pericardial cyst that is not causing compression of the surrounding structures. The patient was started on aspirin and atorvastatin for symptoms of TIA. He remained admitted for two days and his symptoms did not recur during the course of the hospitalization. The decision was made to discharge the patient with outpatient cardiology follow-up for serial echocardiograms to monitor for possible progression in the size of the cyst.

## Discussion

The clinical course of PCs is commonly benign as patients are mostly asymptomatic. They are usually found incidentally when CXR is performed for other reasons. On CXR, they appear as cystic radiodense lesions near the heart, but the use of X-ray is limited due to its inability to differentiate PCs from a pericardial diverticulum. A CT scan is considered superior, as it can delineate the borders, locate the cyst, and differentiate between the types of pericardial lesions. MRI would show the mass as hyperdense on T2-weighted and hypodense on T1-weighted imaging [6]. There are limitations to these modalities, as the protein content of the cystic fluid may cause increased densities on CT scan as well as decreased T2-weighted signals and increased T1-weighted signals on MRI [6]. Transthoracic two-dimensional echocardiography was found to be the superior imaging modality, as it is the least invasive and can pinpoint the exact location of the cyst as well as differentiate it from other potential diagnoses such as a prominent fat pad, left ventricular aneurysm, prominent left atrial appendage, aortic aneurysm, and solid tumors. Color and spectral Doppler can help in differentiating a pericardial cyst from other vascular structures such as a coronary aneurysm [7].

In most cases, PCs go undetected, as they do not cause any symptoms. A patient may experience dyspnea, chest pain, or palpitations in the event the cyst causes cardiac compression or irritation of the nearby structures. Our patient had chest tightness that resolved on its own. Other potential complications include inflammation (pericarditis), cardiac tamponade due to rupture or hemorrhage, and sudden cardiac death [8]. The management of a PC depends on the size of the cyst as well as the patient’s presentation. If the patient is asymptomatic and the cyst is small, serial transthoracic echocardiograms are sufficient. If the patient is symptomatic and/or the cyst is large, resection is considered. Video-assisted thoracoscopic surgery (VATS) is the preferred surgical method. Ideally, intact resection is ultimately the goal, but in the event that the cyst has adhered to the trachea, bronchus, or esophagus, a fragment may be left behind and monitored for recurrence [8]. In cases where the patient is not a candidate for surgery, the more conservative method of ultrasound-guided percutaneous aspiration is considered [5].

## Conclusions

Even though PCs are a rare occurrence, physicians should not neglect to include them in their differentials for patients presenting with chest tightness, shortness of breath, or other symptoms. Through this report, we aim to raise awareness of the importance of further investigations for nonspecific symptoms and differentiating simple pericardial cysts from other pericardial lesions. Based on the symptoms, size, and compression effect of the cyst, management may vary from serial echocardiogram to aspiration or surgical resection.
